# Oral delivery of biologics: from barrier-limited formulation to active convective transport

**DOI:** 10.3389/fddev.2026.1783113

**Published:** 2026-05-04

**Authors:** Sarfaraz K. Niazi

**Affiliations:** Pharmaceutical Sciences, University of Illinois Chicago College of Pharmacy, Chicago, IL, United States

**Keywords:** active delivery systems, barrier-limited epithelial permeability, biologics, convective transport, gastrointestinal barriers, ingestible devices, oral drug delivery, pharmaceutical manufacturing

## Abstract

Biologic therapeutics have transformed the treatment of chronic and life-threatening diseases, yet their clinical use depends almost entirely on parenteral administration. Oral delivery is the preferred route for patients, but it remains challenging for proteins, peptides, and other macromolecules. The gastrointestinal tract blocks their absorption through size exclusion at tight junctions, the hydrophobic lipid bilayer of enterocytes, restricted receptor-mediated uptake, and enzymatic degradation in the lumen and at the brush border. Decades of work on formulation strategies and chemical permeation enhancers have yielded only modest, inconsistent gains. For most biologics, absolute oral bioavailability stays below 1% under physiological conditions. These limitations have prompted a shift from passive transport strategies to active, force-based delivery. Over the past decade, ingestible device platforms have advanced along a clear developmental sequence: early microneedle systems established feasibility in animals, mucus-clearing robotic capsules and liquid-injection auto-injectors extended the approach to liquid biologics in large-animal models, and self-pressurized convective capsules introduced an excipient-driven mechanism requiring no moving parts. This progression culminated in the first Phase 1 human study, which showed that device-based oral delivery of a full-length IgG antibody can match the bioavailability of subcutaneous injection. That result confirms that mechanical bypass of gastrointestinal barriers is achievable in humans and sets a reference point for the field. This review traces the development of active oral biologic delivery systems from early preclinical work to clinical supply, with a focus on convective, force-enabled approaches using self-pressurized capsule technologies. We describe the gastrointestinal barriers that passive strategies cannot overcome, compare device-based platforms by mechanism and evidence, and examine the physical and engineering principles underlying self-pressurized capsule design. We also propose criteria for selecting candidate drugs and address safety, regulatory classification, and manufacturing requirements. The analysis places convective oral delivery within the broader class of force-based platforms and identifies what each system requires to advance.

## 1 Introduction

Biologic drugs, including peptides, proteins, enzymes, antibodies, and nucleic acid-based therapeutics, now account for approximately 40%–50% of pharmaceutical market value (IQVIA Institute, 2023; Mullard, 2023). They represent most newly approved first-in-class therapies and more than half of global blockbuster drug sales (IQVIA Institute, 2023; Mullard, 2023) FDA CDER, 2018–2023. Their clinical value rests on a degree of target specificity and potency that small-molecule drugs rarely achieve.

Three terms used throughout this review require brief definition. Convective force refers to pressure-driven transport of molecules through a medium via bulk fluid motion. Convective transport describes the mass transfer process mediated by that bulk flow. Convective delivery denotes the pharmaceutical application of convective transport to move drugs across biological barriers. These terms are used consistently to distinguish pressure-driven from diffusion-limited mechanisms.

Biologics share a practical limitation: they are not compatible with oral administration. Patients rely on subcutaneous injections, intravenous infusions, or implantable systems. Injection-related pain, needle phobia, sharps disposal, dependence on clinical infrastructure, poor adherence in chronic disease, and limited access in low-resource settings all follow from this requirement (Anselmo and Mitragotri, 2019; Brown et al., 2020).

Oral dosing is convenient, safe, and supports adherence, but the gastrointestinal tract is structured to exclude macromolecules. Tight junction size exclusion, the hydrophobic lipid bilayer of enterocytes, limited receptor-mediated transcytosis, and enzymatic degradation act together to block absorption (Turner, 2009; Shen et al., 2011; Hamman, Enslin and Kotzé, 2005). Epithelial permeability to intact proteins is near zero under normal conditions, and oral bioavailability for most biologics falls below 1%, making conventional oral dosing impractical (Hamman, Enslin and Kotzé, 2005; Moroz, Matoori and Leroux, 2016).

Progress in the field has followed a sequence of increasingly capable approaches. Formulation strategies and chemical permeation enhancers produced real but modest improvements. That experience shifted attention toward active delivery systems that apply physical force to bypass gastrointestinal barriers. Development proceeded from early microneedle proof-of-concept work (Abramson et al., 2019) through mucus-clearing robotic and liquid-injection platforms (Srinivasan et al., 2022; Abramson et al., 2022) and self-pressurized convective capsules (Palacios et al., 2025) to the first human Phase 1 study with the RT-111 RaniPill system (Myers et al., 2026). That study reported 81% relative bioavailability of a full-length IgG versus subcutaneous injection in healthy volunteers, the most advanced clinical result in oral biologic delivery to date.

Self-pressurized oral capsules occupy a distinct position within this field. Effervescence-driven gas generation inside a conventional capsule produces a brief, high-velocity convective pulse that carries drug across the mucus barrier and deposits it at the intestinal epithelium. The system requires no stored mechanical energy, no electronics, and no moving parts, which makes it compatible with standard pharmaceutical manufacturing (Byrne et al., 2021). The OSPRAE capsule, developed by Palacios et al. (2025) at Georgia Tech, is the primary example of this approach. This review examines the broader class of self-pressurized, gas-driven capsule systems using convective oral delivery as its central analytical framework and places each platform within the translational field.

## 2 Physiological barriers to oral delivery of biologics

### 2.1 Chemical and enzymatic degradation in the gastrointestinal tract

Biologics are immediately subject to chemical and enzymatic attack after ingestion. Gastric pH drops below 2, denaturing proteins and accelerating hydrolysis. In the small intestine, trypsin, chymotrypsin, elastase, carboxypeptidases, and brush-border peptidases anchored to enterocytes continue this degradation (Hamman, Enslin and Kotzé, 2005). Between 90% and 99% of orally administered protein may be destroyed before reaching the absorptive epithelium (Hamman, Enslin and Kotzé, 2005; Moroz, Matoori and Leroux, 2016). Enzyme inhibitors reduce degradation but introduce risks of impaired digestion, mucosal irritation, and variable pharmacokinetics (Table 1).

**TABLE 1:** Active oral and ingestible biologic delivery platforms: Chronological development, mechanism, and evidence.

Platform/System	Delivery site	Mechanism	Systemic exposure demonstrated	Development stage	Key references
Permeation enhancers (SNAC, C8, MYCAPSSA)	Gastric/Intestinal surface	Chemical tight-junction modulation	Yes — humans (peptides; no IgG data)	Approved (peptides)	FDA, (2020b); FDA, (2024)
Mucoadhesive patches (chitosan, CAGE ionic liquid)	Intestinal mucosal surface	Extended mucosal residence with local permeation enhancement	No systemic IgG data in humans	Preclinical	Banerjee and Mitragotri (2016)
FcRn-targeted nanocarriers	Epithelial surface (transcytosis)	Receptor-mediated transcytosis of Fc-bearing particles	Preclinical animal data only	Preclinical	Pridgen et al. (2014)
SOMA/LUMI microneedle injector (2019)	Intestinal mucosa (microneedle penetration)	Unfolding arms press microneedle patches against intestinal wall	Preclinical (swine; *ex vivo* human tissue)	Large animal	Abramson et al. (2019)
L-SOMA gastric auto-injector (2022)	Gastric submucosa (needle injection)	Self-righting geometry; needle injection into gastric submucosa	Yes — swine (IgG; up to 80% bioavailability)	Large animal	Abramson et al. (2022)
RoboCap mucus-clearing capsule (2022)	Small intestinal epithelial surface	Rotating capsule clears mucus; payload deposited on exposed epithelium	Preclinical (rodent; 20–40× bioavailability improvement over passive oral)	Preclinical	Srinivasan et al. (2022)
Bioadhesive effervescent foam (2024)	Large intestinal epithelium (topical)	CO_2_-generating foam deposits antibody fragment at colonic mucosa	Preclinical (rodent IBD model)	Preclinical	Zhang et al. (2024)
OSPRAE self-pressurized capsule (2025)	Intestinal epithelial surface (luminal convective plume)	Effervescent gas-driven high-velocity convective ejection; payload deposited near epithelial surface	Preclinical (rodent, insulin; pharmacodynamic effects comparable to SC)	Preclinical	Palacios et al. (2025)
Cephalopod-inspired needle-free microjet (2024)	Intestinal mucosa/submucosa (high-velocity liquid jet)	Compressed gas drives collimated liquid jet into GI wall without solid needles	Yes — large animals (insulin, GLP-1, siRNA)	Large animal	Arrick et al. (2024)
RT-111 RaniPill ingestible injector (2026) ★ CLINICAL	Intestinal submucosa (direct injection)	Ingestible capsule deploys needle and injects into intestinal wall	Yes — humans (IgG biosimilar phase 1; 81% relative bioavailability vs. SC; Tmax 3 days vs. 10 days SC)	Human phase 1	Myers et al. (2026)

Development stage categories: Approved = regulatory approval for indicated use; Human Phase 1 = pharmacokinetic or safety data in humans published; Large animal = systemic exposure demonstrated in swine or comparable large animal; Preclinical = rodent or *ex vivo* data only. The RT-111, human Phase 1 study (Myers et al., 2026) is the only published clinical demonstration of device-based oral IgG delivery. OSPRAE, is included as the representative self-pressurized convective system; the principles described apply to the broader class of effervescence-driven oral delivery systems. ★ denotes the platform that has reached clinical supply.

Chemical stabilization through PEGylation, lipidation, or amino-acid substitution can improve proteolytic resistance. Each approach carries trade-offs: compromised receptor binding affinity (Veronese and Mero, 2008), altered pharmacokinetic profiles (Knudsen et al., 2000), or immunogenic neoepitopes (Sauna et al., 2018). These modifications also increase molecular heterogeneity and manufacturing costs, thereby complicating regulatory approval.

### 2.2 The mucosal barrier: structure, function, and transport limitations

Past the enzymatic environment lies the intestinal mucus barrier, a viscoelastic hydrogel composed primarily of the gel-forming mucin MUC2 secreted by goblet cells throughout the small and large intestine (Johansson et al., 2008; Johansson et al., 2011). It consists of two layers: a firmly adherent inner layer approximately 50–200 μm thick in the human colon, and an outer layer extending an additional 100–700 μm. Total mucus thickness in the small intestine ranges from approximately 100–500 μm, depending on the anatomical region (Atuma et al., 2001).

Mucus is a substantial barrier to drug delivery. Protein diffusivity in intestinal mucus is 10–100-fold lower than in aqueous buffer (Lehr et al., 1992; Lai et al., 2009). Large molecules and nanoparticles move slowly and become trapped by steric hindrance, electrostatic interactions, or hydrophobic binding. The outer mucus layer turns over every 1–4 h (Johansson et al., 2011), while the inner adherent layer is replaced more slowly (Ensign et al., 2012; Lai et al., 2009).

### 2.3 The epithelial cell layer and tight junction regulation

Under the mucus lies the epithelial cell layer, a single sheet of enterocytes joined by tight junctions that regulate paracellular transport. Tight junctions are multiprotein assemblies of claudins, occludin, zonula occludens proteins, and junctional adhesion molecules whose composition varies along the crypt-villus axis (Turner, 2009; Shen et al., 2011). Their effective pore radius is measured in angstroms to a few nanometers, far smaller than any intact biologic.

Attempts to transiently open tight junctions with chemical permeation enhancers have produced modest permeability gains, but clinical translation has been limited by formulation complexity, narrow absorption windows, and variable bioavailability. Some programs have reached approval: oral semaglutide (Rybelsus®) and MYCAPSSA are marketed products, and the SNAC-based candidate MK-0616 is in Phase III development, confirming that chemical enhancement can meet regulatory requirements (Muheem et al., 2016; Moroz, Matoori and Leroux, 2016; Maher et al., 2016). Even so, barrier-limited epithelial permeability keeps achievable bioavailability below 1%–2% for most biologics.

## 3 The active delivery field: a historical developmental arc

The past decade brought rapid development of ingestible systems for gastrointestinal biologic delivery. Progress moved through a clear sequence: preclinical proof-of-concept, large-animal validation, and, most recently, human clinical study. The gap between passive and active approaches is quantifiable. Nanoparticle-based systems reach absolute bioavailabilities of 0.1%–2% for most biologics (Muheem et al., 2016; Moroz et al., 2016); SNAC-formulated oral semaglutide achieves 0.4%–1% (Overgaard et al., 2021). Device-based systems that mechanically bypass the epithelial barrier have achieved substantially higher systemic exposure, with the RT-111 Phase 1 study as the current upper bound (Table 1).

### 3.1 First generation: microneedle and luminal unfolding systems (2019)

The first ingestible biologic delivery devices established that mechanical penetration of the gastrointestinal mucosa is achievable in living animals. The SOMA (Self-Orienting Millimeter-scale Applicator), reported by Abramson et al. (2019), uses asymmetric mass distribution to align with the gastric wall, then deploys drug-loaded milli-posts into the gastric submucosa. In the same report, the LUMI (Luminal Unfolding Microneedle Injector) unfolds arms to press microneedle patches against the small intestinal wall, with feasibility demonstrated in swine and *ex vivo* human tissue. Both systems confirmed that mechanical mucosal penetration can result in measurable systemic macromolecular absorption. At that stage, evidence was limited to preclinical models, and payloads were restricted to solid or semisolid forms.

### 3.2 Second generation: liquid injection and mucus-clearing systems (2022)

Two 2022 platforms extended the first-generation work in different directions. The L-SOMA, the liquid-injecting successor to SOMA, adapted the gastric auto-injector concept to liquid formulations and delivered full-length IgG antibodies into the gastric submucosa of swine with absolute bioavailability of up to 80% (Abramson et al., 2022). That was the first evidence that an ingestible device could achieve systemic IgG levels approaching those of subcutaneous injection in a large-animal model.

The RoboCap system (Srinivasan et al., 2022) took a different approach. A rotating capsule geometry mechanically clears the mucus layer and deposits the payload on the exposed epithelial surface, without penetrating tissue. Bioavailability for insulin and vancomycin improved 20–40-fold over conventional oral delivery in rodent and swine models. Those results showed that mucus clearance alone can produce meaningful absorption gains.

### 3.3 Third generation: convective and CO_2_-Driven systems (2024–2025)

The third generation introduced excipient-driven gas generation as the source of delivery force, eliminating needles and rotating elements. The OSPRAE (Oral Self-Pressurized Aerosol) capsule, developed by Palacios et al. (2025) in the Prausnitz laboratory at Georgia Tech, uses a sodium bicarbonate-citric acid effervescent reaction to build internal pressure. When pressure reaches the failure threshold of a calibrated orifice, a high-velocity gas and drug plume is released at 10–50 m s^-1^ toward the intestinal epithelial surface. In preclinical rodent studies, pressurized delivery at 100–170 kPa drove nanoparticles deep into the mucus barrier. The fraction of payload within 30 μm of the epithelial cell layer increased by an order of magnitude compared with passive deposition, and pharmacodynamic outcomes for insulin matched subcutaneous injection (Palacios et al., 2025) (Table 2).

**TABLE 2:** Transport mechanism limitations and representative references.

Strategy	Transport mechanism	Key limitations	Representative references
Passive transport	Concentration-driven (barrier-limited)	Near-zero epithelial permeability for macromolecules	Brown et al., 2020; Turner, 2009
Chemical enhancers	Tight-junction modulation	Diffuse epithelial risk; narrow absorption window	Muheem et al., 2016; Maher et al., 2016
Nanoparticles	Mucus penetration	Mucus entrapment; low systemic bioavailability	Ensign et al. (2012)
Microneedles/Robotic injection	Mechanical insertion into mucosa	Device complexity; orientation dependence	Abramson et al. (2019)
Convective delivery	Gas-driven bulk transport	Localized, transient tissue interaction; orientation-dependent	Palacios et al. (2025)

UV-crosslinked gelatin may require separate safety justification beyond standard monographs. The lacquer coating used in prototype devices must be replaced with approved enteric polymers (e.g., eudragit, HPMCAS) for clinical use. All materials require biocompatibility assessment under ISO, 10993 standards appropriate for GI, contact.

Zhang et al. (2024) provided independent validation of effervescent chemistry as a delivery force. They demonstrated a rectally administered *in situ* bioadhesive foam for colonic delivery of an anti-TNF-α antibody fragment (Fab) in an inflammatory bowel disease model. CO_2_ generation simultaneously expanded the formulation into foam and acted as a transient permeation enhancer. Rectal Fab foam produced higher plasma Fab concentrations than a solution at a comparable dose and showed therapeutic activity across colitis mouse models. This confirmed that the same gas-generating chemistry can be adapted across different GI segments and delivery modes.

### 3.4 Clinical milestone: the RT-111 RaniPill system (2026)

The most advanced clinical result in the field comes from the RT-111 RaniPill platform, a compressed-gas-driven ingestible injector that deploys a needle within the intestinal wall. Myers et al. (2026) reported 81% relative bioavailability of a full-length ustekinumab biosimilar versus subcutaneous injection in a Phase 1 human study (ClinicalTrials.gov NCT05890118), with no serious adverse events. This is the first published human pharmacokinetic study demonstrating that device-based oral delivery of a full-length IgG can match subcutaneous injection.

The pharmacokinetic profile from the RT-111 study carries broader implications. RT-111 produced a Tmax of 3 days versus 10 days for subcutaneous injection. The slow subcutaneous Tmax reflects lymphatic transit from the hypodermal depot; the faster RT-111 Tmax is consistent with direct vascular entry following intestinal wall penetration. The study did not include thoracic duct cannulation or lymphatic-versus-portal sampling, so the absorption pathway has not been experimentally confirmed. The RT-111 Phase 1 data nonetheless establish a clinical benchmark for the field.

### 3.5 Comparative platform assessment

Each active oral delivery platform has distinct trade-offs. No single technology is superior across all applications. Table 2 provides a chronological comparison of all platforms, from approved permeation enhancers through the RT-111 human Phase 1 data.

### 3.6 Lymphatic routing as an emerging pharmacokinetic consideration

One aspect of pharmacokinetics has received little attention across active delivery platforms: the route by which absorbed drug reaches systemic circulation. Microneedle injectors, jet systems, and convective capsules alike produce portal venous absorption, resulting in hepatic first-pass exposure equivalent to that of conventional oral dosing. This has not been a deliberate design choice; it reflects how oral pharmacology has historically been modeled.

The small intestine contains an extensive lymphatic network, the lacteals, which run parallel to the portal vasculature and drain into the thoracic duct. Lymphatic absorption bypasses hepatic first-pass entirely. In subcutaneous pharmacokinetics, macromolecules above approximately 10 nm in effective diameter preferentially enter lymphatic over blood capillaries from interstitial depots, because lymphatic capillary junctions (100–500 nm) are far wider than blood capillary fenestrations (6–12 nm) (Richter and Jacobsen, 2014; Supersaxo et al., 1990; McLennan et al., 2005). For subcutaneous IgG, the established dominant mechanism is lymphatic transport from the hypodermal interstitium.

Whether intramucosal deposition of appropriately formulated macromolecules can result in preferential lymphatic drainage from the jejunal lamina propria remains an open question. No published study has directly measured vascular versus lymphatic partitioning of any molecule above 50 kDa deposited at this site. The question is testable by standard thoracic duct cannulation. Convective delivery systems that deposit the drug into the lamina propria interstitium, rather than onto the luminal surface, create the anatomical conditions under which preferential lymphatic entry could occur. If it does, hepatic first-pass elimination would be reduced or eliminated, and the systemic distribution pattern would more closely resemble subcutaneous injection. This question has direct implications for the design trajectory of active oral delivery systems.

## 4 Limitations of barrier-limited transport and chemical enhancement strategies

**FIGURE 1:**
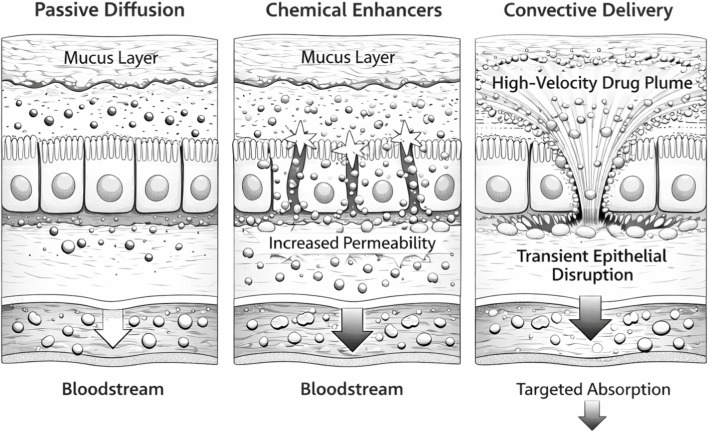
Schematic comparison of drug transport mechanisms across the intestinal epithelium, contrasting passive diffusion-limited approaches with active force-based delivery systems. Convective delivery bypasses the structural and biological barriers that constrain passive transport (Brown et al., 2020; Anselmo and Mitragotri, 2019) (Drawn by BioRender).

Most oral biologic delivery technologies share an underlying premise: that incremental improvements in epithelial transport will eventually produce useful absorption. This premise has shaped nanoparticle engineering, muco-penetrating coatings, enzyme inhibitors, and permeation enhancers for decades (Figure 1). The dominant barriers, tight junction size exclusion, lipid bilayer impermeability, limited transcytosis, and enzymatic degradation, are structural and biological rather than diffusional. Formulation optimization cannot overcome them.

Clinical data support this conclusion. Oral semaglutide (Rybelsus®), the most successful oral peptide formulation approved to date, achieves approximately 0.4%–1% bioavailability in human pharmacokinetic studies despite extensive optimization (Overgaard et al., 2021). Its SNAC absorption enhancer transiently raises local gastric pH and promotes transcellular absorption of semaglutide across the gastric epithelium (Buckley et al., 2018). The product requires strict fasting, reflecting the narrow, fragile absorption window of chemically enhanced transport. Even with full formulation optimization, barrier-limited permeability caps what is achievable.

Convective transport, the movement of material by bulk fluid or gas flow, has been applied for decades in jet injection and microneedle-assisted delivery. Self-pressurized oral capsules bring this principle into a standard pharmaceutical format. Convective force can be generated *in situ* using simple excipients within a conventional capsule architecture, with burst pressure reproducibility of ±15% CV at 100–170 kPa (Palacios et al., 2025). This reframes oral biologic delivery as a controlled physical transport problem rather than a permeability problem (Figure 1). Repeat-dose safety data and chronic inflammatory biomarker assessments have not yet been established.

## 5 Convective force as a transport mechanism in the gastrointestinal tract

### 5.1 Physical basis of convective delivery: *Ex Vivo* and preclinical evidence

Two physical barriers govern convective transport in the gastrointestinal tract: the mucus layer and the epithelial cell layer. Intestinal mucus is a viscoelastic, non-Newtonian hydrogel (Taylor et al., 2003; Macierzanka et al., 2011). Its resistance to penetration falls under sufficiently high localized force because of its shear-thinning properties. The epithelial cell layer can tolerate brief, localized mechanical disruptions and undergoes rapid restitution afterward (Feil et al., 1987; Feil et al., 1989; Lacy, 1988).

In the convective delivery model, a brief, high-velocity gas plume displaces mucus through momentum transfer, creating a transient crater that exposes the epithelial surface. In *ex vivo* porcine intestine, pressurized delivery at 100–170 kPa drove nanoparticles deep into the mucus barrier and increased the fraction of payload within 30 μm of the epithelial cell layer by an order of magnitude compared with passive deposition (Palacios et al., 2025). The 30 μm zone corresponds to the firmly adherent inner mucus layer, which turns over slowly and retains deposited material longer, improving the opportunity for epithelial uptake (Figure 1).

### 5.2 Distinction between convective force and ballistic penetration

Convective oral delivery does not depend on ballistic penetration by drug particles (Byrne et al., 2021; Mitragotri, 2004). The OSPRAE mechanism uses a gas-driven convective front to clear fluid and mucus from the delivery path. Drug particles are carried in the moving gas stream and deposited at or near the epithelial surface; they are not projected as projectiles. This has practical consequences for safety and scalability. Payload size (20–1,000 nm in reported studies) does not substantially affect delivery efficiency across the tested pressure range, so different payload types can be used without redesigning the platform.

### 5.3 Self-pressurization as an enabler of oral convective delivery

The key practical contribution of the convective delivery approach is the generation of the required force *in situ* from pharmaceutical excipients, without stored mechanical energy (Byrne et al., 2021; Sung et al., 2014). The reaction of sodium bicarbonate and citric acid in water produces CO_2_ in a controllable and pressurizable manner. This requires no electronics, batteries, or moving parts. The capsule remains inert during manufacturing, storage, and gastric transit, and activates only upon contact with intestinal fluid. Compatibility with standard pharmaceutical production and disposal follows directly from this design.

Effervescent CO_2_ generation has been applied independently in the OSPRAE convective capsule (Palacios et al., 2025) and in bioadhesive foam systems for colonic delivery (Zhang et al., 2024), confirming the pharmaceutical tractability of this chemistry across platforms. The same gas-generating reaction can drive capsule rupture, foam expansion, or mucoadhesive depot formation, depending on the system’s design.

### 5.4 Pharmacokinetic implications of convective intestinal delivery

Insulin delivered to the intestinal epithelium via self-pressurized capsules produces rapid reductions in blood glucose with kinetics comparable to those of subcutaneous injection, despite lower systemic insulin exposure (Edgerton et al., 2006; Moore et al., 2012). The explanation lies in intestinal physiology: insulin absorbed from the small intestine enters the portal circulation and reaches the liver before it reaches the systemic circulation. This portal-first pharmacology resembles endogenous insulin secretion more closely than subcutaneous injection does and may carry metabolic advantages beyond route convenience.

**FIGURE 2:**
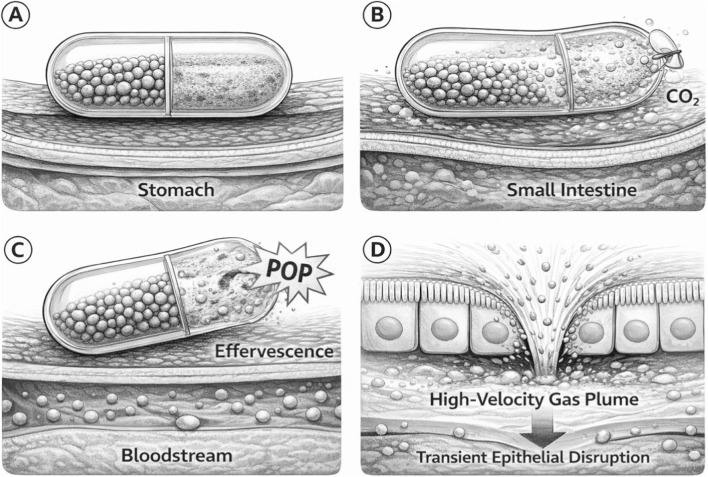
Self-pressurized oral capsule delivery sequence. **(A)** Intact capsule during gastric transit. **(B)** CO_2_ generation triggered by intestinal fluid. **(C)** Internal pressurization and orifice failure. **(D)** High-velocity drug plume deposited at the epithelial surface (Palacios et al., 2025). Drawn by BioRender.

This portal-first advantage applies to all active oral delivery systems that deposit the drug into the small intestinal wall. It is distinct from lymphatic routing, which would bypass the liver entirely. In principle, the two mechanisms are complementary: a fraction of payload could be absorbed via the portal vein, producing a rapid onset and hepatic targeting, while another fraction enters the lymphatics with a delayed onset and no hepatic first-pass, depending on formulation properties and depot-site anatomy (Figure 2).

### 5.5 Platform generalization

Convective force is a generalizable transport mechanism, not a drug-specific workaround (Brown et al., 2020; Anselmo and Mitragotri, 2019). Because the driving force is physical, convective delivery is independent of molecular structure, receptor affinity, or enzymatic susceptibility at the point of transport. Drug candidates can therefore be selected by therapeutic relevance and dose requirements rather than by permeability, a criterion that does not apply to force-driven transport.

## 6 Engineering principles, design attributes, and translational challenges

Active force-based oral delivery systems differ from passive formulation approaches in how they bypass biological barriers. This section examines engineering attributes and failure modes shared across convective capsule systems, mucus-clearing robotic devices, and needle-free jet systems, drawing on the systems described in Table 2. Specific published data are cited where available; attributes that apply broadly across platforms are presented as class properties. The goal is a practical engineering and quality framework for any active oral delivery program.

### 6.1 Payload containment and protection of labile biologics

All active oral delivery systems face the same primary challenge: preserving the integrity of a fragile macromolecule through manufacturing, storage, and gastrointestinal transit until delivery. In convective capsule systems, the drug and the effervescent excipients occupy separate compartments connected by a porous interface. CO_2_ passes freely while the drug stays contained until burst-driven entrainment (Palacios et al., 2025). In the L-SOMA, the drug sits in a liquid reservoir sealed from gastric fluid by a multilayer capsule architecture and is released only after self-orientation and actuation (Abramson et al., 2022). In robotic mucus-clearing systems, the payload is isolated from the luminal environment during mucus removal and deposited topically on the exposed epithelium (Srinivasan et al., 2022). In every case, the payload compartment must withstand gastric acid, luminal enzymes, and mechanical agitation while releasing on a defined trigger.

**FIGURE 3:**
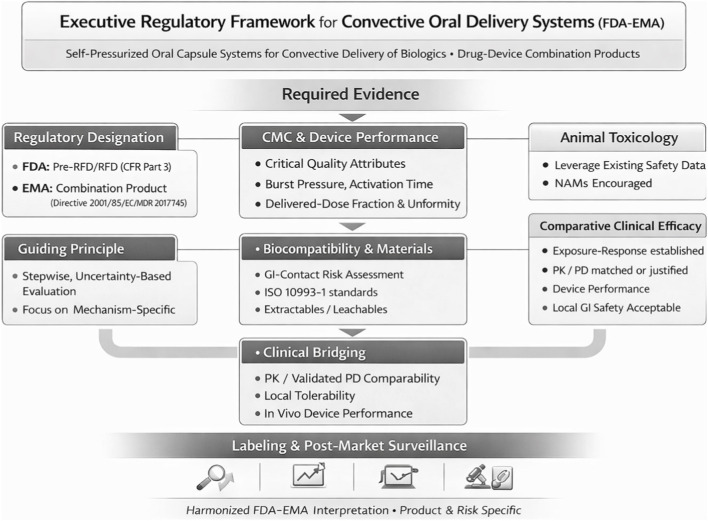
Proposed regulatory pathway for convective oral drug-device combination products (FDA, 2019; FDA, 2023b). Drawn by BioRender.

Drug content uniformity is a shared critical quality attribute across all platforms. Target specifications require 90%–110% of label claim with a relative standard deviation below 6%, consistent with ICH Q6B. This applies to liquid reservoir filling in injection-based systems and to dry-powder or lyophilized payloads in convective systems (Palacios et al., 2025; Abramson et al., 2022; Dhalla et al., 2022) (Figure 3).

### 6.2 Capsule shell integrity, material selection, and gastric protection

Every ingestible delivery system needs an outer shell that survives gastric transit intact and triggers in the proximal small intestine. In self-pressurized effervescent systems, the shell must withstand internal pressurization before controlled rupture, which requires reinforcement beyond standard capsule specifications. UV crosslinking of gelatin increases mechanical strength and enzymatic resistance while preserving eventual degradability (Palacios et al., 2025). In liquid-injection systems such as L-SOMA and RT-111, the capsule houses a spring or gas reservoir and must stay watertight through gastric transit until actuation, relying on multilayer enteric coatings with defined pH-dissolution thresholds (Abramson et al., 2022; Myers et al., 2026). In jet systems, the capsule must hold compressed gas or spring elements without premature release (Arrick et al., 2024).

Across all platforms, prototype materials must be replaced with pharmacopeial accepted components before clinical use. UV-crosslinked gelatin may require separate biocompatibility justification beyond standard monographs. Prototype coatings, lacquers, experimental polymers, and non-standard adhesives must give way to enteric polymers with established regulatory status, such as Eudragit or HPMCAS. All materials that contact the gastrointestinal mucosa or the systemic circulation require biocompatibility assessment under ISO 10993-1 for GI-contact devices (FDA, 2020a).

### 6.3 Controlled actuation and deterministic failure mechanics

When and how force is applied is the central engineering challenge for all active delivery systems. In convective capsule systems, a calibrated delivery orifice and a zone of selectively reduced wall thickness or coating serve as the designed failure site. Capsule rupture occurs predictably at a defined pressure and location (Palacios et al., 2025). Orifice diameter (0.5–2.0 mm) and shell crosslinking density together determine the burst pressure, which is tunable to 30–170 kPa in the OSPRAE system. The failure mode is elastic-to-plastic: the orifice deforms outward before rupture, reducing fragmentation risk. This is analogous to pressure-relief design used in industrial vessels and microelectromechanical systems.

In needle-based and jet-based systems, actuation depends on spring release, compressed gas discharge, or a staged mechanical sequence triggered by pH, temperature, or humidity. The L-SOMA uses a two-spring mechanism that first extends a needle then injects the payload. Timing is critical: premature or incomplete actuation delivers drug into the lumen rather than tissue (Abramson et al., 2022). In cephalopod-inspired jet systems, compressed CO_2_ drives a collimated liquid jet; the pressure profile governs penetration depth and delivered-dose fraction (Arrick et al., 2024). In the RoboCap system, motor-driven rotation clears mucus; rotational speed and timing determine how much mucus is removed before payload release (Srinivasan et al., 2022). Across all platforms, actuation must remain reproducible despite variability in GI transit time, pH, fluid volume, and motility.

### 6.4 Gas-generating excipient chemistry and its cross-platform applications

Effervescent reactions between bicarbonate salts and organic acids offer a practical route to generating mechanical force inside the gastrointestinal tract without external power or stored energy. In self-pressurized convective capsules, this reaction builds pressure until the orifice failure threshold is reached, releasing a high-velocity gas-drug plume that penetrates the mucus layer. Bench testing shows burst-pressure reproducibility with a coefficient of variation of ±15%, and *ex vivo* measurements record delivery velocities of 10–50 m s^-1^ at the orifice exit (Palacios et al., 2025).

Zhang et al. (2024) applied the same chemistry in a different format: a rectally administered *in situ* bioadhesive foam for colonic delivery of an anti-TNF-α antibody fragment to treat inflammatory bowel disease. Potassium bicarbonate and citric acid react on contact with rectal fluid, producing CO_2_ that simultaneously aerates the formulation into foam and transiently disrupts the colonic mucus and epithelial barrier. In a non-everted rat gut *ex vivo* model, a single exposure improved macromolecule transepithelial flux by more than tenfold. *In vivo*, rectal Fab foam produced higher plasma Fab concentrations than a Fab solution at comparable dose and showed therapeutic activity in acute and chronic colitis mouse models. The independent application of effervescent chemistry across convective capsules, foam-based colonic delivery, and other gas-driven systems confirms CO_2_ generation as a broadly applicable excipient-based force delivery strategy.

### 6.5 Device orientation, mucosal apposition, and delivered-dose efficiency

Device orientation at the time of force application is an unsolved engineering problem shared by all active delivery platforms. The consequences differ by mechanism. In needle-injection systems, incorrect orientation sends the needle into the lumen rather than tissue, with complete dose loss. In convective plume systems, the drug is ejected into the luminal fluid rather than toward the epithelium. In mucus-clearing systems, the rotating element fails to contact the mucosal surface.

Several engineering solutions have been developed. Asymmetric mass distribution exploits gravity to orient the device toward the dependent wall (L-SOMA, SOMA). Self-righting geometry based on the leopard tortoise allows reorientation from any starting position (Abramson et al., 2019; Abramson et al., 2022). Multi-orifice or radially symmetric jet configurations deliver force in all directions, reducing orientation dependence (Arrick et al., 2024). Mucoadhesive anchor elements fix the device to the wall before actuation. Density-asymmetric capsule designs address orientation in convective systems (Palacios et al., 2025). Rough estimates suggest a single fixed orifice achieves correct mucosal apposition in one-quarter to one-third of delivery events; multi-orifice or asymmetric designs may raise this to one-half to two-thirds. These estimates require empirical confirmation in physiologically relevant intestinal models before they can be used as design specifications.

Delivered-dose fraction, the share of nominal payload reaching the epithelial surface rather than luminal fluid, capsule remnants, or shed mucus, is uncharacterized for all active delivery platforms under physiologically relevant conditions, including fed versus fasted state, variable motility, and mucosal disease. Quantitative mass-balance studies using radiolabeled tracers, fluorescent nanoparticle payloads, and pharmacokinetic deconvolution are required for any IND-enabling development program.

### 6.6 Critical quality attributes for active oral delivery systems

Critical quality attributes for active oral delivery systems cover both device performance and drug product quality, and must be defined in alignment with ICH Q8, Q9, and Q10 (FDA, 2012). Table 3 lists CQAs applicable across the platform classes in this review, with target ranges drawn from published data where available.

**TABLE 3:** Critical quality attributes for active oral biologic delivery systems.

CQA	Description	Target/Specification	Source
Gastric protection integrity	Shell remains intact during gastric transit	100% intact after 2 h at pH 1.2 (enteric-coated systems)	Palacios et al. (2025), Abramson et al. (2022)
Actuation trigger reliability	Activation within defined window following GI transit to target segment	15–60 min post-gastric emptying for small-intestinal sites	All active platforms
Force generation reproducibility	Burst pressure (convective); jet velocity (microjet); needle penetration depth (injection)	100–170 kPa ±15% CV (convective); validated specification ranges (other systems)	Palacios et al. (2025), Arrick et al. (2024)
Delivery geometry	Plume velocity and duration (convective); injection depth (needle)	10–50 m s^-1^ at orifice exit; 10–100 m plume duration (convective)	Palacios et al. (2025)
Drug content uniformity	Label claim accuracy across filled units	90%–110% label claim; RSD <6% (ICH Q6B)	ICH Q6B; Palacios et al. (2025)
Delivered-dose fraction	Fraction of nominal payload reaching epithelial surface or tissue	≥50% within 100 μm of epithelium (convective); ≥80% into tissue (needle)	Palacios et al. (2025), Abramson et al. (2022)
Needle actuation completeness	Confirmation that needle extends fully and retracts (injection systems only)	No retained hardware; confirmed by radiographic or sensor data	Myers et al. (2026), Abramson et al. (2022)

### 6.7 Failure mode and effects analysis

Table 4 presents a cross-platform failure mode and effects analysis covering failure modes common to the major categories of active oral delivery systems. Severity rankings are qualitative. Formal Risk Priority Number calculations should be performed during product-specific development in accordance with ICH Q9 (ICH, 2005).

**TABLE 4:** Failure mode and effects analysis for active oral biologic delivery systems (cross-platform).

Failure mode	Potential Cause(s)	Potential Effect(s)	Severity	Affected platforms	Risk mitigation
Premature gastric rupture	Insufficient shell reinforcement; inadequate enteric protection	Loss of dose; gastric irritation; potential mucosal injury	High	All platforms	Enteric coating validation; stress testing at simulated gastric conditions; stability studies
Incomplete actuation/Partial pressurization	Moisture ingress; excipient degradation; orifice blockage; spring failure	Insufficient force; high dose variability; lumen-side delivery	Moderate–High	Convective, injection, jet	Moisture-barrier packaging; excipient quality specifications; actuation confirmation
Unfavorable device orientation	Random capsule positioning in GI lumen	Payload ejected into lumen rather than tissue; reduced dose efficiency	Moderate–High	All platforms	Self-orienting geometry; multi-orifice design; density asymmetry; mucoadhesive anchor elements
Excessive tissue trauma/Perforation	Over-pressurization; incorrect depth calibration	Epithelial injury; inflammation; perforation risk at high pressures	High	All platforms	Pressure-limiting design; smooth fracture failure mode; depth-limiting needle stops; pre-use pressure validation
Device fragment retention	Brittle materials; over crosslinking; mechanical overload	Retained foreign body; mucosal irritation; obstruction risk	Low–Moderate	All platforms	Material selection for ductility and biodegradability; radiopacity markers for clinical monitoring
Payload aggregation or denaturation	Mechanical shear during ejection; temperature excursion; premature hydration	Loss of potency; immunogenicity risk; dose reduction	High	Convective, jet	Protective excipient formulation; stress testing across actuation conditions; post-ejection potency assays
Failure to reach target GI segment	Rapid GI transit; disease-altered motility; capsule retention in stomach	No delivery at intended site; loss of pharmacological effect	Moderate	All platforms	Enteric coating pH thresholds validated for target segment; transit-time studies in animal models

Severity rankings are qualitative across all platform classes. Formal Risk Priority Number (RPN) calculations and platform-specific FMEA, refinement should be performed during product development in accordance with ICH Q9.

### 6.8 Tissue interaction, safety profile, and epithelial recovery

The safety of force-based oral delivery depends on the degree, depth, and reversibility of tissue disruption at the delivery site. Histological evaluations across multiple active delivery platforms consistently show disruptions confined to areas smaller than approximately 1 mm^2^, with preservation of surrounding villi, crypt architecture, and submucosal integrity (Abramson et al., 2019; Byrne et al., 2021; Palacios et al., 2025). Disruptions affect the apical epithelial surface rather than the deeper regenerative compartments. Epithelial restitution follows within minutes to hours through migration of adjacent cells, a proliferation-independent repair mechanism that operates continuously in healthy intestinal tissue (Feil et al., 1987; Feil et al., 1989; Lacy, 1988). No persistent ulceration, crypt damage, or inflammatory infiltration has been reported following pressurized delivery.

Jet-based systems produce disruption profiles that vary with jet velocity, nozzle diameter, and fluid viscosity. At the pressures used in the Arrick et al. (2024) cephalopod-inspired system, tissue penetration was sufficient for systemic macromolecule absorption in large animals with no evidence of perforation. Needle-based systems such as L-SOMA and RT-111 produce puncture sites of defined diameter and depth; the RT-111 Phase 1 study reported device remnant clearance by Day 3 in all participants with no serious adverse events (Myers et al., 2026). RoboCap produces surface abrasion from mucus clearing rather than deep penetration, with no mucosal injury in preclinical models (Srinivasan et al., 2022). Bioadhesive foam systems cause epithelial contact without mechanical disruption; colitis mouse models showed no additional mucosal injury from the formulation (Zhang et al., 2024).

A direct comparison of the barrier-compromise area, duration, and cumulative effects between convective delivery and chemical enhancers has not been performed. This comparison is mechanistically plausible but experimentally unconfirmed. Chronic safety data for repeat dosing are absent for all active delivery platforms. Chronic-use indications will require repeat-dose GI toxicology studies of at least 6 months in rodents and 9–12 months in non-rodent species, with endpoints including serial histopathology, inflammatory biomarkers, barrier permeability markers, endotoxin levels, and microbiome composition (Tables 3, 4).

### 6.9 Manufacturing complexity, scalability, and regulatory classification

Active oral delivery systems differ substantially in manufacturing complexity. Needle-based and robotic systems with spring assemblies, electronics, or precision microneedle arrays require micron-scale tolerances and multi-step assembly, which are difficult to scale for high-volume pharmaceutical production. Retained hardware, electronics, or non-biodegradable components add regulatory complexity beyond standard combination product requirements, and may require additional biocompatibility characterization, device testing under ISO 11135/11607, or post-market surveillance for device residues.

Self-pressurized convective capsule systems are at the simpler end of this spectrum. Gelatin shell, effervescent excipients, and standard coating materials are individually conventional and available from established pharmaceutical suppliers at a commodity scale. The manufacturing steps, UV crosslinking, selective orifice formation, and dual-compartment filling, operate within millimeter-scale tolerances achievable with modified standard capsule-filling equipment (Palacios et al., 2025). Effervescent foam systems (Zhang et al., 2024) require only formulation-level process control, with no device assembly required.

All active oral delivery systems will be classified as drug-device combination products requiring formal designation by the FDA Office of Combination Products (21 CFR Part 3). Lead center assignment, CDER or CDRH, depends on the primary mode of action and requires a formal Pre-Request for Designation interaction. IND-enabling packages must include device performance characterization, biocompatibility data, and targeted GI safety studies alongside standard drug development requirements (FDA, 2019; FDA, 2020a).

### 6.10 Critical appraisal: limitations and translational barriers

The evidence base for all active oral delivery platforms shares structural limitations. *In vivo* pharmacodynamic data come from a small number of model drugs, predominantly insulin and, more recently, IgG, in a small number of species. Extension to other biologics, other patient populations, and the full range of human GI physiology will require broad validation. The most advanced human clinical data come from the RT-111 injection system (Myers et al., 2026). No convective or robotic platform has yet published human pharmacokinetic data.

Capsule orientation under realistic GI conditions, including variable luminal diameter, peristaltic contraction, fluid volume, and luminal contents, is incompletely characterized across platforms. The delivered-dose fraction and its variability under fed versus fasted conditions, altered GI motility, and mucosal disease states are not established. Regulatory pathways have not been formally defined through agency interaction for any force-based platform covered in this review, other than the RT-111 program.

Active delivery is not appropriate for all drugs. Poor candidates include drugs requiring daily doses above 50–100 mg, molecules with narrow therapeutic windows where delivery variability would be harmful, and agents susceptible to mechanical shear or aggregation during force-driven ejection. Conditions associated with disrupted mucosal architecture, altered mucus properties, or unpredictable GI transit, including active inflammatory bowel disease, radiation enteritis, and short bowel syndrome, may reduce delivery efficiency or raise local safety risk. These exclusion criteria apply to the platform class.

## 7 Rational selection of target drugs for convective oral delivery

### 7.1 Reframing target selection beyond permeability

Conventional oral drug development starts with a permeability screen: molecules that fail Caco-2 or PAMPA assays are dropped early. Convective oral delivery reverses this logic. Transport is driven by physical force, not molecular permeability, so intrinsic permeability no longer determines candidacy. Selection is guided instead by dose feasibility, pharmacodynamic potency, absorption-site relevance, and therapeutic index (Palacios et al., 2025; Anselmo and Mitragotri, 2019).

### 7.2 Candidate drug selection criteria matrix

Table 5 presents a scoring framework for prioritizing candidate drugs. Total score ≥20 indicates high-priority candidates; 12–19 moderate-priority; below 12 low-priority or unsuitable. Scores are illustrative and require validation against empirical delivery data.

**TABLE 5:** Candidate drug selection criteria matrix for convective oral delivery.

Criterion	Weight	Favorable (2)	Acceptable (1)	Unfavorable (0)	Rationale/Reference
Therapeutic dose	3	≤5 mg	5–20 mg	>20 mg	Capsule volume constraint; Palacios et al., 2025
Therapeutic index	3	TI >10	TI 5–10	TI <5	Pharmacological risk; Muller and Milton, 2012
PD response steepness	2	Steep	Moderate	Flat	Steep dose-response tolerates delivery variability
Portal-first advantage	2	Significant hepatic benefit	Partial benefit	None	Edgerton et al. (2006), Moore et al. (2012)
Administration burden	2	Daily/frequent injection	Weekly injection	Monthly or less	Higher burden = greater patient benefit
Dry-powder stability	1	>24 months	12–24 months	<12 months	Supply chain/shelf-life requirements
Local GI toxicity risk	2	Minimal	Moderate/manageable	High	Epithelial-contact safety consideration

### 7.3 Protein and peptide therapeutics: Prime candidates

Table 6 lists drug classes best suited to convective oral delivery based on the selection framework. Full-length IgG antibodies represent the highest unmet need. The RT-111 Phase 1 data (Myers et al., 2026) confirm that bioavailability comparable to subcutaneous injection is achievable for this class (Tables 5, 6).

**TABLE 6:** Drug classes prioritized for convective oral delivery.

Drug class	Examples	Current route	Rationale/Key references
Metabolic hormones	Insulin, GLP-1 analogues	SC injection	High potency; portal-first benefit; Palacios et al., 2025; Overgaard et al., 2021
Bone regulators	PTH, calcitonin	Injection	Intermittent dosing; Brown et al., 2020
Cytokines	IL-10, interferons	Injection	μg-level potency; Anselmo and Mitragotri, 2019
Growth factors	FGF21, EPO fragments	Injection	Low dose requirements; Cao et al., 2021
Full-length IgG antibodies	Ustekinumab, adalimumab analogues	SC/IV injection	Highest unmet need: RT-111 phase 1 data confirm feasibility; Myers et al., 2026

### 7.4 Non-protein drugs with historically poor oral bioavailability

The platform also applies to small molecules that fail oral delivery due to extensive first-pass metabolism, rapid luminal degradation, or solubility-limited absorption. Dry-powder or lyophilized formulations can be entrained in the delivery plume (Brown et al., 2020).

### 7.5 Drugs unsuitable for convective oral delivery

Drugs are unsuitable when they require daily doses above 50–100 mg, have a narrow therapeutic window in which delivery variability poses clinical risk, cause local tissue toxicity on mucosal contact, or require continuous rather than pulsed systemic exposure.

## 8 Regulatory classification and pathway considerations

No direct regulatory precedent exists for effervescence-driven oral drug-device combination products. The requirements described below are anticipated based on analogous products and existing frameworks. Formal agency interaction is required to establish the precise expectations for any specific platform.

### 8.1 Combination product classification: United States

Force-based oral delivery systems combine a pharmacologically active substance with a mechanical delivery mechanism, placing them within combination product regulation. In the United States, the Office of Combination Products assigns primary regulatory oversight based on the product’s primary mode of action (FDA, 2019). For platforms where therapeutic effect is mediated by the drug, the primary mode of action is typically pharmacological, pointing to lead review by CDER or, for biologics, CBER. Assignment proceeds under 21 CFR Part 3 (FDA, 2005). Sponsors should initiate the Pre-RFD/RFD process early and must not assume combination product classification without formal agency interaction (FDA, 2023a).

Core IND-enabling expectations include CMC and device performance data covering burst pressure, activation time, plume characteristics, and delivered-dose fraction; biocompatibility per ISO 10993-1 (FDA, 2020a); and targeted nonclinical GI safety evaluation. Routine systemic animal toxicology may be waivable when the biologic has an established systemic safety profile, and risk is primarily local or mechanical, consistent with the FDA Modernization Act 2.0 (U.S. Congress, 2022). Comparative efficacy trials may be unnecessary when exposure-response relationships are established, and PK/PD profiles are matched (FDA, 2023b). Manufacturing requirements for combination products follow cGMP regulations, which require harmonization of drug and device quality systems (FDA, 2017). Pre-IND or Type B meetings are advisable before submitting the IND (FDA, 2023b).

For IgG antibody payloads, the RT-111 Phase 1 study (Myers et al., 2026) has advanced the regulatory landscape by confirming that the FDA engages with ingestible drug-device combination products targeting the intestinal wall and that a regulatory framework for this product class exists. Specific requirements still depend on formal Pre-RFD interaction.

### 8.2 European Union framework

In the European Union, these products are regulated under the EMA framework for medicinal products incorporating medical devices, as integral drug-device combinations in which the medicinal product constitutes the primary mode of action (EMA, 2021). Device components must comply with the Medical Device Regulation (EU) 2017/745, including conformity assessment and, where required, notified body involvement (European Commission, 2017). The EMA guideline on quality requirements for drug-device combinations sets out expectations for device characterization, performance testing, and integration into the medicinal product dossier (EMA, 2021).

### 8.3 International harmonization and ICH framework

At the international level, ICH Q8 (pharmaceutical development), Q9 (quality risk management), and Q10 (pharmaceutical quality systems) provide the overarching quality framework for development and lifecycle management of complex products (ICH, 2009a; ICH, 2005; ICH, 2009b). These guidelines call for risk-based approaches, design space characterization, and integration of product and process understanding, all of which apply directly to combination systems in which device performance affects drug delivery and clinical outcomes.

### 8.4 Risk-benefit considerations and regulatory maturity

Regulatory uncertainty around force-based oral delivery reflects early field maturation, not a development block. FDA, EMA, and ICH frameworks provide a working foundation, but applying them to these platforms requires interpretation and, in some areas, extension. Until standardized evaluation paradigms are established, including validated *in vitro* and *in vivo* delivery performance models and defined bioequivalence criteria, regulatory review will proceed on a case-by-case basis. Iterative engagement between sponsors and regulatory agencies will be necessary to build precedents for future programs.

For patients requiring lifelong injections, modest risks of localized, transient epithelial disruption may be acceptable when weighed against improved adherence and quality of life. Portal-first pharmacokinetics may translate into better long-term outcomes for certain drug classes (Edgerton et al., 2006; Moore et al., 2012). The risk-benefit calculation is indication-specific, depends on the therapeutic index, and must be assessed formally in clinical development.

## 9 Commercial manufacturing, cost of goods, and global accessibility

### 9.1 Manufacturing philosophy

The OSPRAE platform adds delivery functionality without a proportional increase in manufacturing complexity (Byrne et al., 2021; Sheskey et al., 2020). Compared with microneedle or robotic capsule systems, self-pressurized convective capsules require no micron-scale fabrication, no electronics assembly, and no sterile component handling beyond standard biologic filling.

### 9.2 Unit operations and scalability

Manufacturing involves capsule shell preparation with UV crosslinking, external coating, selective orifice formation, excipient filling, drug compartment fabrication, and final assembly. None of these steps requires micron-scale alignment (Palacios et al., 2025). Table 7 lists the materials and their regulatory status.

**TABLE 7:** Materials used in self-pressurized oral capsules and regulatory status.

Component	Material	Regulatory status	References
Capsule shell	Gelatin	GRAS/Pharmacopeial	Sheskey et al. (2020)
Gas generator	NaHCO_3_, citric acid	Widely approved	Nykanen et al. (2004)
Drug matrix	MCC, PEO	Approved excipients	Vanza et al. (2020)
Coating (prototype)	Lacquer (replaceable)	Replaceable with approved enteric polymer (Eudragit, HPMCAS)	Palacios et al. (2025)

### 9.3 Materials cost, cost of goods, and global accessibility

All materials are available at metric-ton scale from multiple global suppliers at costs far below those of precision-machined device components. Convective oral delivery eliminates the need for sterile injection infrastructure, reduces dependence on the cold chain, and simplifies packaging, comparable to conventional oral solid dosage forms. The platform suits biosimilar developers, lifecycle management programs, and global health initiatives, particularly in settings where injectable biologics are impractical due to infrastructure limitations.

## 10 Future directions and platform expansion

The most immediate applications for convective oral delivery are reformulations of currently injectable biologics with favorable dose-potency profiles, established systemic safety records, and chronic injection burdens that reduce patient compliance. GLP-1 agonists, insulin, PTH, and selected cytokines are the leading candidates.

Expansion opportunities include capsule miniaturization for pediatric and geriatric patients, enteric targeting for regional delivery, multi-compartment architectures for sequential payload release, and extension to nucleic acid therapeutics (mRNA, ASOs, siRNA), live biologics, bacteriophages, and microbiome-modulating agents (Anselmo and Mitragotri, 2019; Byrne et al., 2021).

An open question relevant to both convective and injection-based systems is whether formulation design can shift post-deposition absorption from portal to lymphatic routing. As discussed in Section 3.6, size-dependent interstitial partitioning suggests that appropriately sized lipid-associated particles deposited in the lamina propria interstitium could preferentially enter lacteals rather than blood capillaries, thereby eliminating hepatic first-pass. Thoracic duct cannulation experiments in lamina propria-injection models are a near-term experimental priority that could redirect delivery system design across platforms.

Digital health integration can correlate delivery events with patient-specific physiological states and add monitoring tools without modifying the capsule. Platform evolution should be guided by target engagement, potency, and safety, not by incremental improvement of passive transport within constraints that the active delivery paradigm has already replaced (Table 7).

## 11 Conclusion

Oral delivery of biologics has been treated as an unsolved problem for decades, limited by the structural and biological barriers of the gastrointestinal tract. Strategies that work within those barriers have produced incremental progress without changing the fundamental picture. The past decade altered this by establishing a clear developmental arc: multiple ingestible platforms demonstrated systemic macromolecular absorption in animal models, and the RT-111 Phase 1 data (Myers et al., 2026) confirmed that device-based oral IgG delivery at clinically meaningful levels is achievable in humans. A first milestone of clinical supply has been reached.

Self-pressurized oral capsules offer a distinct approach within this field. Simple pharmaceutical excipients combined with engineered mechanical failure produce transient, localized convective delivery events that bypass biological barriers without complex devices, electronics, or invasive hardware. Preclinical evidence shows effective delivery of fragile biologics, such as insulin, with pharmacodynamic outcomes that match injection and tissue disruption, well within the repair capacity of the intestinal epithelium. Critical knowledge gaps remain in the quantitative characterization of delivered-dose fraction and mass balance; orientation effects on delivery probability; chronic safety under repeat dosing; barrier function impacts beyond acute epithelial repair; and clinical validation of preclinical pharmacodynamic findings.

Beyond convective delivery, the field’s most consequential open question is whether intramucosal deposition combined with appropriate formulation design can redirect absorption from the portal vasculature to the intestinal lymphatics. An affirmative answer would move the field from barrier bypass to deliberate pharmacokinetic control of the oral absorption route. Answering it requires direct experimental measurement in relevant animal models and does not depend on any specific device platform.

Convective oral delivery has the potential to change how drugs are administered, designed, selected, and deployed. By shifting the focus from molecular permeability to physical transport, this platform extends the reach of oral dosing to biologics previously confined to needles and infusion lines.
